# Genomic alterations in the WNT/β-catenin pathway and resistance of colorectal cancer cells to pathway-targeting therapies

**DOI:** 10.37349/etat.2025.1002295

**Published:** 2025-02-25

**Authors:** Ioannis A. Voutsadakis

**Affiliations:** IRCCS Istituto Romagnolo per lo Studio dei Tumori (IRST) “Dino Amadori”, Italy; ^1^Algoma District Cancer Program, Sault Area Hospital, Sault Ste. Marie, ON P6B 0A8, Canada; ^2^Section of Internal Medicine, Division of Clinical Sciences, Northern Ontario School of Medicine, Sudbury, ON P3E 2C6, Canada

**Keywords:** Canonical WNT pathway, *RNF43*, *CTNNB1*, *TCF7L2*, treatment resistance

## Abstract

**Aim:**

Colorectal cancer is the most prevalent gastrointestinal malignancy with limited therapeutic options in the metastatic setting. The WNT/β-catenin/adenomatous polyposis coli (APC) pathway is commonly deregulated in the disease and presents a rational target for therapeutic exploitation.

**Methods:**

The publicly available genomic data from the colorectal cancer cohort of the Cancer Genome Atlas (TCGA) were used to define groups of colorectal cancers with alterations in *APC* or other key genes of the WNT/β-catenin/APC pathway and to identify genomic characteristics of interest in each group. In vitro sensitivity data for drugs targeting the pathway were compiled from the Genomics of Drug Sensitivity in Cancer (GDSC) project.

**Results:**

Three-fourths of colorectal cancers possessed *APC* alterations and about one in four of these cases possessed also concomitant alterations in other genes of the WNT/β-catenin/APC pathway, including *RNF43*, *CTNNB1*, and *TCF7L2*. Colorectal cancers with alterations in one or more of the three genes of the WNT/β-catenin pathway, *RNF43*, *CTNNB1*, and *TCF7L2*, in the absence of *APC* alterations, were frequently microsatellite instability (MSI) high and had high tumor mutation burden (TMB). Cancers with these same alterations in the three genes with or without *APC* alterations presented a high frequency of mutations in receptor tyrosine kinases, PI3K/AKT pathway genes, and DNA damage response genes. Cell lines without mutations in WNT/β-catenin/APC pathway components displayed numerically greater sensitivity to inhibitors of the pathway in vitro.

**Conclusions:**

Groups of colorectal cancers differing in WNT/β-catenin/APC pathway alterations present diverse genomic landscapes that could have therapeutic implications for the rational development of inhibitors of the pathway.

## Introduction

Colorectal cancer represents the most prevalent gastrointestinal malignancy in Western countries and one of the most lethal neoplasms globally [1, 2]. Metastatic disease can be effectively palliated by chemotherapy treatments, which may also prolong the survival of responding patients [3]. Moreover, progress in the genomic characterization of colorectal cancers has led to effective targeted therapies for specific sub-sets of colorectal cancers possessing targetable alterations [4–7]. For example, checkpoint inhibitor immunotherapy is effective in mismatch repair (MMR) deficient colorectal cancers [4]. Colorectal cancers with V600E *BRAF* mutations may be treated with combinations of small molecule *BRAF* inhibitors and anti-EGFR monoclonal antibodies and the less common HER2 over-expressing colorectal cancers may be treated with drugs targeting this alteration [5, 6]. The majority of metastatic colorectal cancer patients who bear MMR proficient tumors or tumors with *KRAS* mutations (besides *KRAS G12C*) have few options available besides chemotherapy and anti-angiogenic agents [8].

Besides the receptor tyrosine kinase/KRAS/BRAF/MEK pathway, other pathways frequently deregulated in colorectal cancers include p53, the TGFβ/SMAD cascade, and the WNT/β-catenin/adenomatous polyposis coli (APC) pathway, which is most commonly activated through mutations in tumor suppressor *APC* [9]. All these frequently altered pathways are currently not targeted clinically but represent potential therapeutic opportunities in colorectal cancer given their prevalence and pathogenic involvement in the disease [10, 11]. Inhibition of the aberrantly activated WNT/β-catenin/APC pathway could be the most attractive therapeutic target, as mutations of *APC* are present in up to three-fourths of colorectal cancers and remaining cases have common mutations in other components of the pathway [12, 13]. *APC* is a regulator of the assembly of the β-catenin destruction complex, which promotes β-catenin phosphorylation and ubiquitination and leads to its proteasome degradation [14]. Mutations in *APC* promote β-catenin stabilization and signaling even in the absence of external signals for the activation of the pathway [15]. Other mutations activating the WNT/β-catenin/APC pathway occur less frequently in colorectal cancers, but they may also produce aberrant activation. These include activating mutations in *CTNNB1* gene encoding for β-catenin, inactivating mutations of ubiquitin ligase RNF43, which is a regulator of the abundance of Frizzled receptors of the pathway, and activating mutations of *TCF7L2*, encoding for the β-catenin transcription co-factor TCF4 [12]. In the current investigation, the landscapes of colorectal cancers with mutations in the WNT/β-catenin/APC pathway are elucidated and therapeutic implications are explored based on in vitro cell line data, as well as knock down and knock out arrays.

## Materials and methods

### Colorectal cancer cohort

Clinical and genomic data of patients with colorectal cancer were extracted from the colorectal cancer cohort of the Cancer Genome Atlas (TCGA) [12]. The cohort includes a total of 594 patients, of whom 534 patients were profiled for mutations. TCGA used whole exome sequencing for the genomic analyses. In addition to mutations, TCGA provided analyses of copy number alterations and structural variants. Single nucleotide mutation calling was conducted with input from various pipelines [16]. Copy number alterations were analyzed in TCGA studies with the GISTIC (Genomic Identification of Significant Targets in Cancer) algorithm, which assigns a score of 2 or above in genes with putative amplification [17]. For quantification of chromosomal instability (CIN), TCGA used a score (aneuploidy score, AS) which was calculated in each sample as the sum of the number of chromosome arms in the sample that had copy number gains or losses. For the calculation of AS, chromosome arms were defined as copy number altered if more than 80% of their length contained somatic copy number alterations. In contrast, chromosome arms with somatic copy number alterations extending from 20% to 80% of their length were considered indeterminate and chromosome arms with somatic copy number alterations in less than 20% of their length were considered not altered. For the calculation of the AS from Affymetrix 6.0 SNP arrays an algorithm called ABSOLUTE was used in TCGA [18].

### Colorectal cancer cell lines

The Cancer Cell Line Encyclopedia (CCLE) is an extensive collection of cancer cell lines procured from various agencies and analyzed using whole exome sequencing [19]. Similar to TCGA, the determination of copy number alterations in the CCLE collection was performed with the GISTIC algorithm [17]. Data on drug sensitivity of cell lines from colorectal cancer with APC and other WNT/β-catenin pathway mutations were obtained from the Genomics of Drug Sensitivity in Cancer (GDSC) dataset (http://www.cancerrxgene.org) [20]. The GDSC project contains two experimental datasets, GDSC1 and GDSC2, that differ in the experimental conditions used. GDSC1 experiments were performed between 2009 and 2015. These experiments used media alone in the negative control cell lines not exposed to drugs. The most recent dataset GDSC2 performed after 2015 and used in the current study employed media with vehicle (DMSO, dimethylsulfoxide) in the negative controls. Dependencies on knock down or knock out of specific genes in colorectal cancer cell lines with APC and other WNT/β-catenin pathway mutations were obtained from the DepMap portal that contains data from RNA interference (RNAi) arrays and CRISPR (Clustered Regularly Interspaced Short Palindromic Repeats) arrays of cell lines from the CCLE collection [21, 22]. CRISPR and RNAi arrays identify essential genes that are important for the survival of assayed cell lines and, as a result, the absence of these essential genes has a significant survival and proliferation effect in vitro [23–25]. The genes and dependencies discovered with the two array methodologies do not completely overlap, as the depth of suppression differs. RNAi experiments in DepMap were from project Achilles which used the DEMETER algorithm for analysis [24]. CRISPR arrays in DepMap were from project SCORE containing 323 cancer cell lines from various cancers and a library of 18,009 targeted genes [26]. Computational modeling of experiments in SCORE was performed with the CERES and the CHRONOS algorithms [27, 28].

Interrogation of both TCGA cohort and colorectal cancer cell lines at the individual case and cell line level was performed online at the cBioPortal for Cancer Genomics platform (cBioportal, http://www.cbioportal.org). cBioportal is a site containing genomic and associated clinical data from publicly available studies, maintained by Memorial Sloan Kettering Cancer Center (MSKCC) and other academic institutions [29, 30]. Two groups of alterations in the WNT/β-catenin pathway, the first consisting of *APC* alterations and the second consisting of alterations in three other WNT/β-catenin pathway genes (*RNF43*, *CTNNB1*, and *TCF7L2*) were considered in the TCGA cohort and groups of patients were constructed in a 2 by 2 manner resulting in a total of four groups.

Statistical comparisons of categorical and continuous data were carried out with the Fisher exact test or the *χ*^2^-test and the *t*-test or ANOVA. All statistical comparisons were considered significant if *P* < 0.05.

## Results

### Prevalence and clinical comparison in the four groups

The frequently altered colorectal cancer tumor suppressor, *APC* showed alterations (most commonly mutations and a small number of deletions) in 395 of the 534 cases (74%) in the colorectal cancer cohort of TCGA with mutation data. Of the *APC* altered cases, 95 cases (17.8% of the entire cohort) possessed also concomitant alterations (mostly mutations and a small number of amplifications or deletions) in one or more of three other WNT/β-catenin pathway genes, *RNF43*, *CTNNB1*, and *TCF7L2* (termed quadruple altered cohort) and 300 cases (56.2% of the entire cohort) had no alterations in these three genes (termed *APC* only altered cohort). Colorectal cancers without *APC* alterations constituted 26% of the cases in the colorectal cancer TCGA cohort and among them, 44 cases (8.2% of the entire cohort) had alterations in *RNF43*, *CTNNB1*, or *TCF7L2* (termed triple altered cohort) and 95 cases (17.8% of the entire cohort) had no alterations in *RNF43*, *CTNNB1*, and *TCF7L2* (quadruple wild type cohort). The four groups did not differ significantly in their average age, percentage of older (above 65 years old) patients, the distribution of sexes, or the stage of colorectal cancer at diagnosis (Table 1). The majority of cancers in the triple altered cohort (93.2%) were located in the colon and only 6.8% of those cancers were rectal, while in the three other groups, 25% to 30% of the cases were rectal (*χ*^2^-test *P* = 0.01, Table 1). Significant differences were also observed between the groups in their genomic characteristics (Table 2). The triple altered group had a majority (60%) of microsatellite instability (MSI) cases, which were less frequent in the other cohorts (22% in the quadruple altered and quadruple wild type cohorts and only 3.2% in the *APC* only altered cohort). Chromosome instability (CIN), on the other hand, was more prevalent in the *APC* only altered cohort (83% of cases), followed by the quadruple wild type cohort (66% of cases) and the quadruple altered cohort (53.5% of cases), while the triple altered group presented the lowest CIN prevalence (35% of cases). Consistent with the genomic sub-type prevalence, the triple altered group had the highest frequency of high tumor mutation burden (TMB) (above 10 mutations/Mb) and the highest frequency of low AS and FGA scores compared with the three other groups (Table 2).

**Table 1:** Characteristics of colorectal cancers with *APC*, *RNF43*, *CTNNB1*, or *TCF7L2* alterations from the Cancer Genome Atlas (TCGA)

**Characteristic**	**Entire cohort (*n* = 594)**	**Quadruple altered (*n* = 95)**	***APC* only altered (*n* = 300)**	**Triple altered (*n* = 44)**	**Quadruple wild type (*n* = 95)**	***P***
Age (mean ± SD)	66.1 ± 13.4	64.4 ± 13	66.2 ± 11.9	65.8 ± 16.5	65.7 ± 14.3	0.72
Age						
≤ 65 years-old	260 (43.9)	48 (51.1)	134 (44.8)	18 (40.9)	40 (42.1)	0.56
> 65 years-old	332 (56.1)	46 (48.9)	165 (55.2)	26 (59.1)	55 (57.9)	
NA	2	1	1	0	0	-
Sex						
Male	312 (52.7)	47 (50)	164 (54.8)	23 (52.3)	43 (45.3)	0.41
Female	280 (47.3)	47 (50)	135 (45.2)	21 (47.7)	52 (54.7)	
NA	2	1	1	0	0	-
Stage						
I	104 (17.9)	13 (14.4)	59 (20.1)	6 (13.6)	17 (18.3)	I–II versus III–IV: 0.26
II	220 (37.9)	39 (43.3)	98 (33.5)	23 (52.3)	41 (44.1)	
III	170 (29.3)	23 (25.6)	97 (33.1)	12 (27.3)	19 (20.4)	
IV	86 (14.8)	15 (16.7)	39 (13.3)	3 (6.8)	16 (17.2)	
NA	14	5	7	0	2	-
Location primary						
Colon	436 (74.1)	70 (75.3)	209 (70.4)	41 (93.2)	71 (75.5)	0.01
Rectal	152 (25.9)	23 (24.7)	88 (29.6)	3 (6.8)	23 (24.5)	
NA	6	2	3	0	1	-

Percentages are shown in parentheses. NA: not available; SD: standard deviation; APC: adenomatous polyposis coli. -: no data

**Table 2:** Subtype, tumor mutation burden (TMB), aneuploidy score (AS), and fraction genome altered (FGA) in colorectal cancers with or without APC and other WNT/β-catenin pathway alterations from the Cancer Genome Atlas (TCGA)

**Characteristic**	**Entire cohort (*n* = 594)**	**Quadruple altered (*n* = 95)**	***APC* only altered (*n* = 300)**	**Triple altered (*n* = 44)**	**Quadruple wild type (*n* = 95)**	***P***
Subtype						
GS	58 (12.7)	11 (12.7)	39 (13.8)	2 (5)	6 (12)	GS versus CIN versus MSI: < 0.00001
CIN	328 (71.4)	46 (53.5)	235 (83)	14 (35)	33 (66)	
MSI	63 (13.7)	19 (22.1)	9 (3.2)	24 (60)	11 (22)	
POLE	10 (2.2)	10 (11.7)	0	0	0	-
NA	135	9	17	4	45	-
TMB						
≤ 10 mutations/Mb	451 (84.5)	63 (66.3)	288 (96)	17 (38.6)	83 (87.4)	< 0.00001
> 10 mutations/Mb	83 (15.5)	32 (33.7)	12 (4)	27 (61.4)	12 (12.6)	
NA	60	0	0	0	0	-
AS						
< 4	108 (18.4)	30 (32.3)	39 (13.1)	29 (65.9)	15 (15.8)	< 0.00001
4–24	427 (72.9)	58 (62.4)	226 (76.1)	15 (34.1)	70 (73.7)	
> 24	51 (8.7)	5 (5.3)	32 (10.8)	0	10 (10.5)	
NA	8	2	3	0	0	-
FGA						
< 0.075	115 (19.7)	28 (30.1)	32 (10.9)	24 (55.8)	16 (17)	< 0.00001
0.075–0.35	319 (54.7)	49 (52.7)	179 (61.1)	15 (34.9)	54 (57.5)	
> 0.35	149 (25.6)	16 (17.2)	82 (28)	4 (9.3)	24 (25.5)	
NA	11	2	7	1	1	-

Percentages are shown in parentheses. GS: genomically stable; CIN: chromosomal instability, MSI: microsatellite instability; NA: not available; POLE: polymerase epsilon. -: no data

### Mutation comparison in the four groups

**Figure 1:**
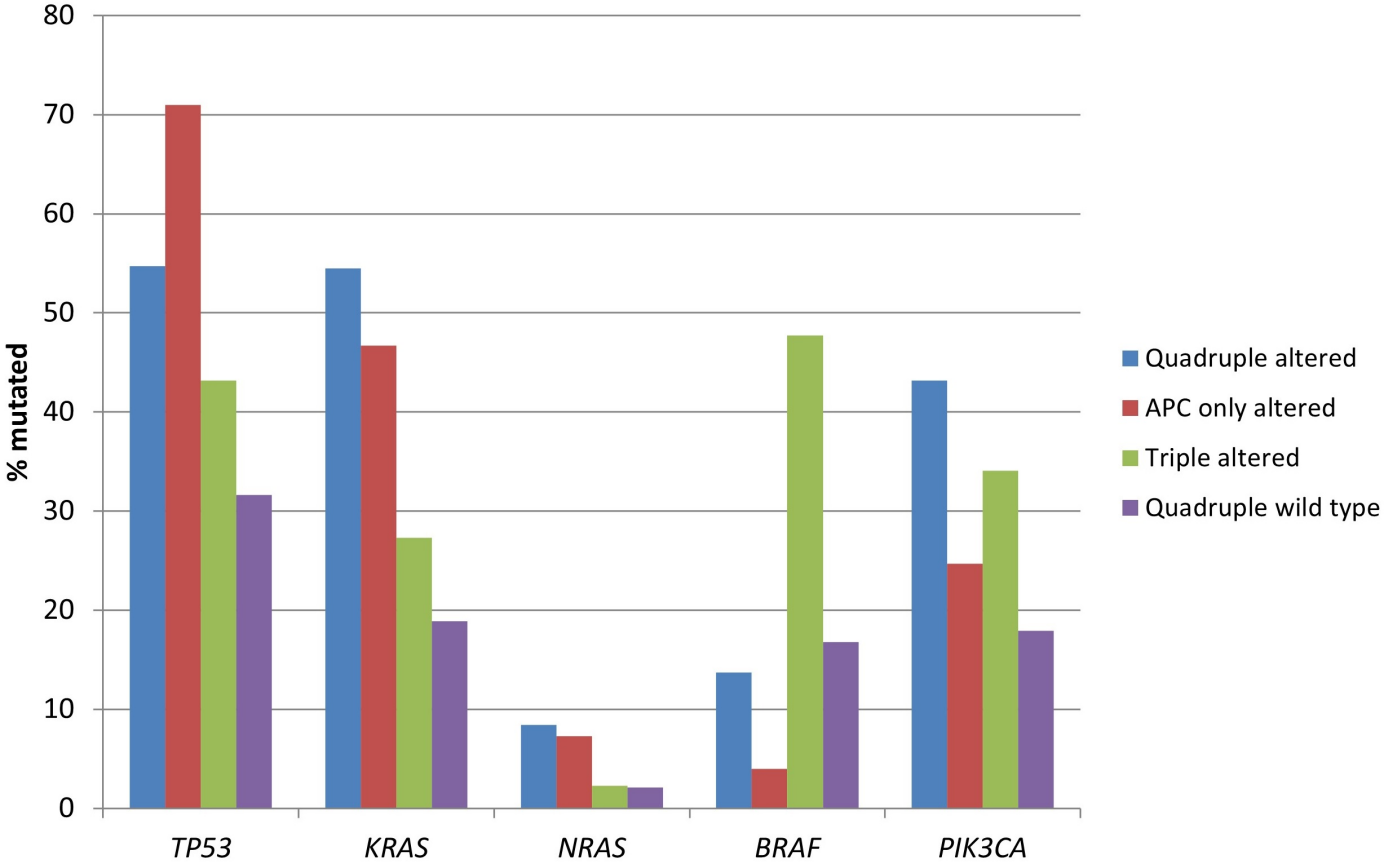
**Prevalence of mutations in frequently mutated cancer-associated genes in colorectal cancers with or without APC and other WNT/β-catenin/APC pathway alterations**. Data are from TCGA (the Cancer Genome Atlas). APC: adenomatous polyposis coli

Mutations in the gene encoding for tumor suppressor p53, *TP53*, were more frequent in the two groups with *APC* alterations (54.7% of cases in the quadruple altered group and 71% of cases in the *APC* only altered group) compared with the two groups without *APC* alterations (43.2% of cases in the triple altered group and 31.6% of cases in the quadruple wild type group, *χ*^2^-test *P* < 0.00001, Figure 1). Mutations in oncogene *KRAS*, and in the less prevalent *NRAS*, were also more frequent in the two groups with *APC* alterations (*χ*^2^-test *P* < 0.00001 for *KRAS*, but not reaching significance, *P* = 0.14, for *NRAS*). *BRAF* mutations were significantly more prevalent in the triple altered group, with 47.7% of cases showing mutations, compared with 4% to 16.8% of cases in the other groups (*χ*^2^-test *P* < 0.00001, Figure 1). *PIK3CA* mutations were more prevalent in the two groups with *RNF43*, *CTNNB1*, or *TCF7L2* alterations (*χ*^2^-test *P* = 0.0003).

**Figure 2:**
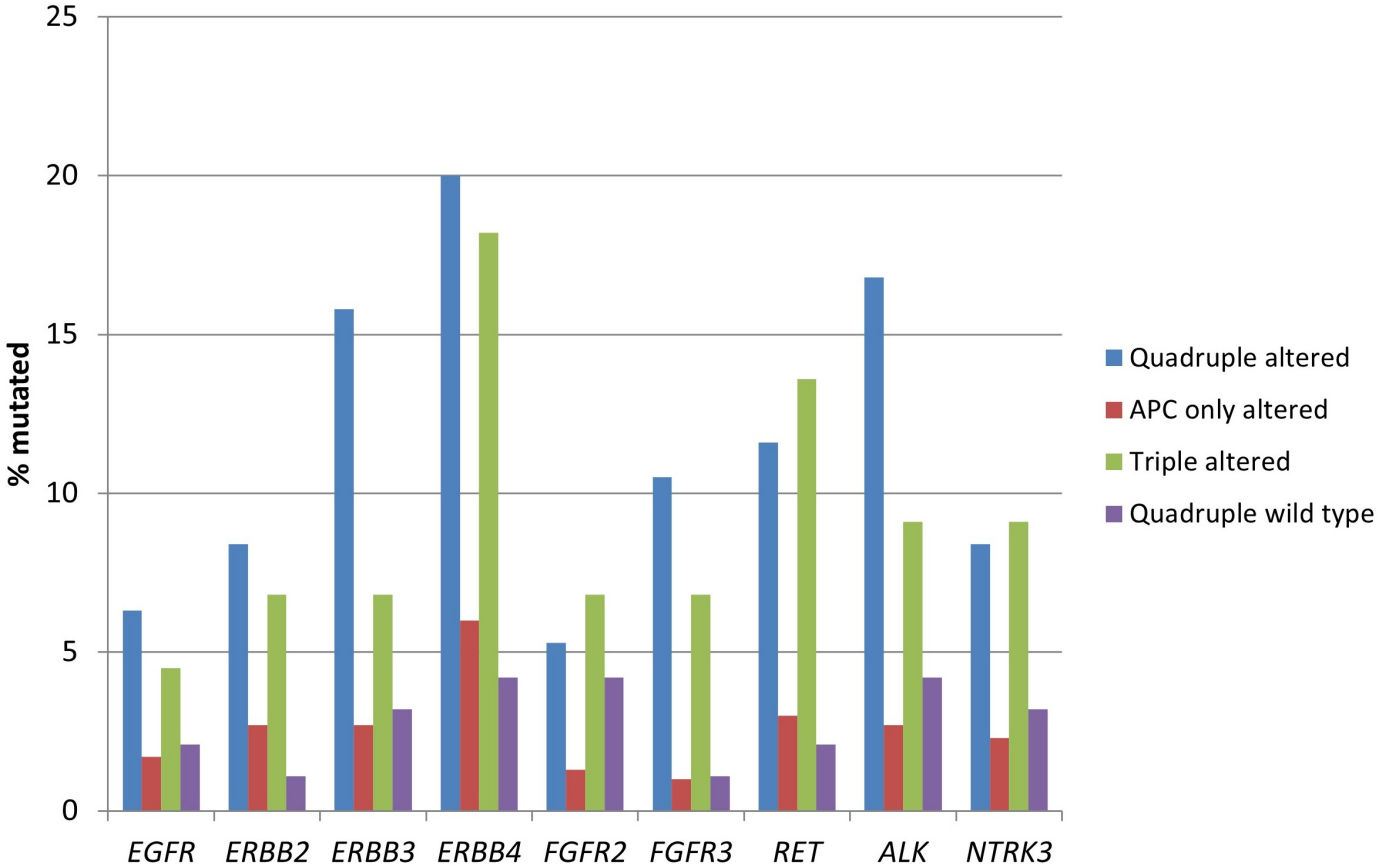
**Prevalence of mutations in receptor tyrosine kinase genes in colorectal cancers with or without APC and other WNT/β-catenin/APC pathway alterations**. Data are from TCGA (the Cancer Genome Atlas). APC: adenomatous polyposis coli

**Figure 3:**
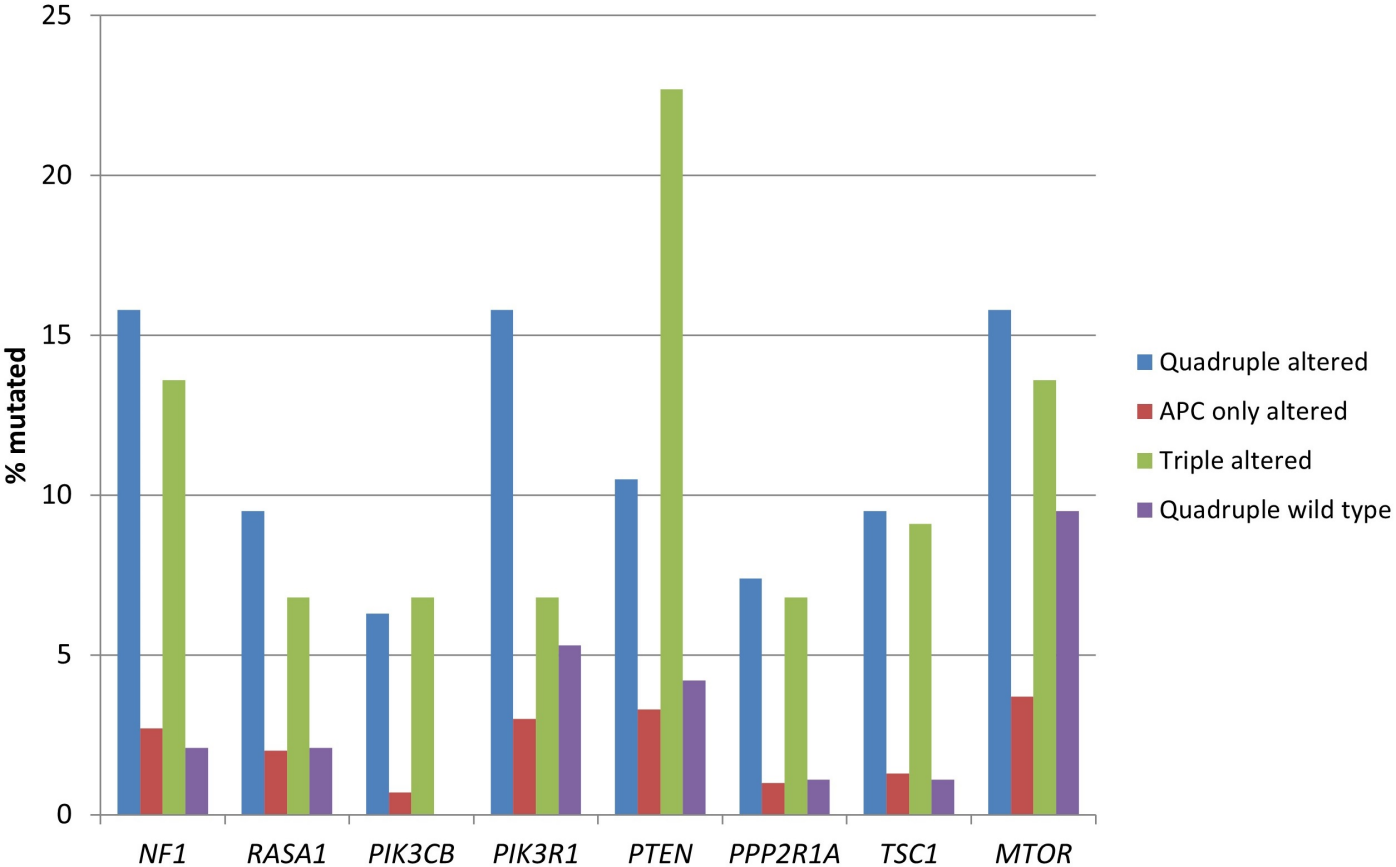
**Prevalence of mutations in genes encoding for proteins of the KRAS/BRAF/MEK and the PI3K/AKT/mTOR pathways in colorectal cancers with or without APC and other WNT/β-catenin/APC pathway alterations**. Data are from TCGA (the Cancer Genome Atlas). APC: adenomatous polyposis coli

The genes encoding for receptor tyrosine kinases, including members of the EGFR family, members of the FGFR family, *RET*, *ALK*, and *NTRK3* were significantly more frequently mutated in the two groups with *RNF43*, *CTNNB1*, or *TCF7L2* alterations (Table 3 and Figure 2). Moreover, several proteins of the KRAS/BRAF/MEK and the PI3K/AKT/mTOR pathways had a higher prevalence in the same groups (Table 3 and Figure 3).

**Table 3:** Mutation frequencies in representative cancer-associated genes in colorectal cancers with or without APC and other WNT/β-catenin pathway alterations from the Cancer Genome Atlas (TCGA)

**Gene**	**Entire cohort (*n* = 534)**	**Quadruple altered (*n* = 95)**	***APC* only altered (*n* = 300)**	**Triple altered (*n* = 44)**	**Quadruple wild type (*n* = 95)**	***P***
*EGFR*	19 (3.6)	6 (6.3)	5 (1.7)	2 (4.5)	2 (2.1)	0.09
*ERBB2*	20 (3.7)	8 (8.4)	8 (2.7)	3 (6.8)	1 (1.1)	0.02
*ERBB3*	32 (6.0)	15 (15.8)	8 (2.7)	3 (6.8)	3 (3.2)	0.00001
*ERBB4*	62 (11.6)	19 (20)	18 (6)	8 (18.2)	4 (4.2)	0.00002
*FGFR2*	22 (4.1)	5 (5.3)	4 (1.3)	3 (6.8)	4 (4.2)	0.06
*FGFR3*	18 (3.4)	10 (10.5)	3 (1)	3 (6.8)	1 (1.1)	0.00002
*RET*	30 (5.6)	11 (11.6)	9 (3)	6 (13.6)	2 (2.1)	0.0003
*ALK*	41 (7.7)	16 (16.8)	8 (2.7)	4 (9.1)	4 (4.2)	< 0.00001
*NTRK3*	29 (5.4)	8 (8.4)	7 (2.3)	4 (9.1)	3 (3.2)	0.02
*NF1*	43 (8.1)	15 (15.8)	8 (2.7)	6 (13.6)	2 (2.1)	< 0.00001
*RASA1*	29 (5.4)	9 (9.5)	6 (2)	3 (6.8)	2 (2.1)	0.004
*PIK3CB*	16 (3.0)	6 (6.3)	2 (0.7)	3 (6.8)	0	0.001
*PIK3R1*	38 (7.1)	15 (15.8)	9 (3)	3 (6.8)	5 (5.3)	0.0001
*PTEN*	48 (9.0)	10 (10.5)	10 (3.3)	10 (22.7)	4 (4.2)	< 0.00001
*PPP2R1A*	15 (2.8)	7 (7.4)	3 (1)	3 (6.8)	1 (1.1)	0.001
*TSC1*	21 (3.9)	9 (9.5)	4 (1.3)	4 (9.1)	1 (1.1)	0.0001
*MTOR*	46 (8.6)	15 (15.8)	11 (3.7)	6 (13.6)	9 (9.5)	0.0003
*MSH2*	27 (5.1)	15 (15.8)	3 (1)	3 (6.8)	0	< 0.00001
*MSH6*	29 (5.4)	14 (14.7)	3 (1)	3 (6.8)	4 (4.2)	< 0.00001
*PMS2*	16 (3.0)	8 (8.4)	4 (1.3)	2 (4.5)	0	0.001
*MLH1*	24 (4.5)	7 (7.4)	5 (1.7)	7 (15.9)	3 (3.2)	0.00004
*POLE*	50 (9.4)	24 (25.3)	6 (2)	3 (6.8)	3 (3.2)	< 0.00001
*BRCA1*	19 (3.6)	9 (9.5)	3 (1)	3 (6.8)	1 (1.1)	0.00009
*BRCA2*	69 (12.9)	19 (20)	12 (4)	6 (13.6)	1 (1.1)	< 0.00001
*ATM*	107 (20.0)	23 (24.2)	25 (8.3)	12 (27.2)	10 (10.5)	0.00002
*BRIP1*	25 (4.7)	11 (11.6)	3 (1)	0	4 (4.2)	0.00002
*CDK12*	42 (7.9)	13 (13.7)	7 (2.3)	8 (18.2)	3 (3.2)	< 0.00001
*LRP1B*	190 (35.6)	29 (30.5)	41 (13.7)	11 (25)	13 (13.7)	0.0007
*AMER1*	72 (13.5)	22 (23.2)	32 (10.7)	6 (13.6)	7 (7.4)	0.004
*AXIN2*	36 (6.7)	10 (10.5)	7 (2.3)	7 (15.9)	5 (5.3)	0.0001
*FAT1*	99 (18.5)	26 (27.4)	13 (4.3)	12 (27.3)	5 (5.3)	< 0.00001
*FAT4*	124 (23.2)	35 (36.8)	54 (18)	21 (47.7)	14 (14.7)	< 0.00001

Percentages are shown in parentheses. APC: adenomatous polyposis coli

**Figure 4:**
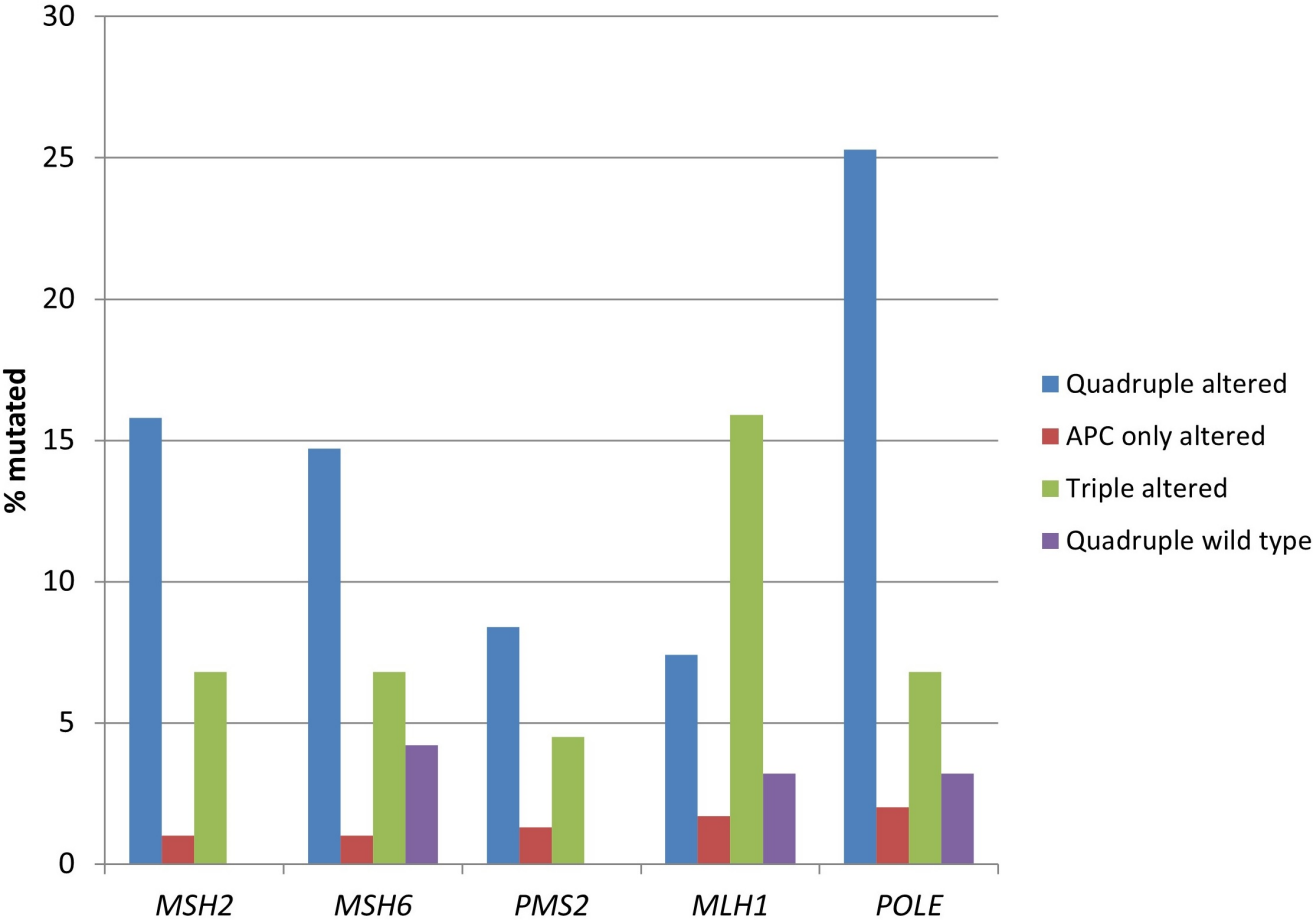
**Prevalence of mutations in mismatch repair associated genes and proofreading polymerase epsilon gene (*POLE*) in colorectal cancers with or without APC and other WNT/β-catenin/APC pathway alterations**. Data are from TCGA (the Cancer Genome Atlas). APC: adenomatous polyposis coli

Consistent with the higher prevalence of MSI and high TMB, the two groups with *RNF43*, *CTNNB1*, or *TCF7L2* alterations possessed statistically significant higher mutation rates of genes encoding for MMR-associated proteins and proofreading polymerase epsilon (Table 3 and Figure 4). The quadruple wild type group showed a low rate of mutations in these genes, despite containing 22% of MSI high cases, suggesting that epigenetic modifications such as *MLH1* promoter methylation may be the responsible alterations involved in producing MMR defects in this group.

**Figure 5:**
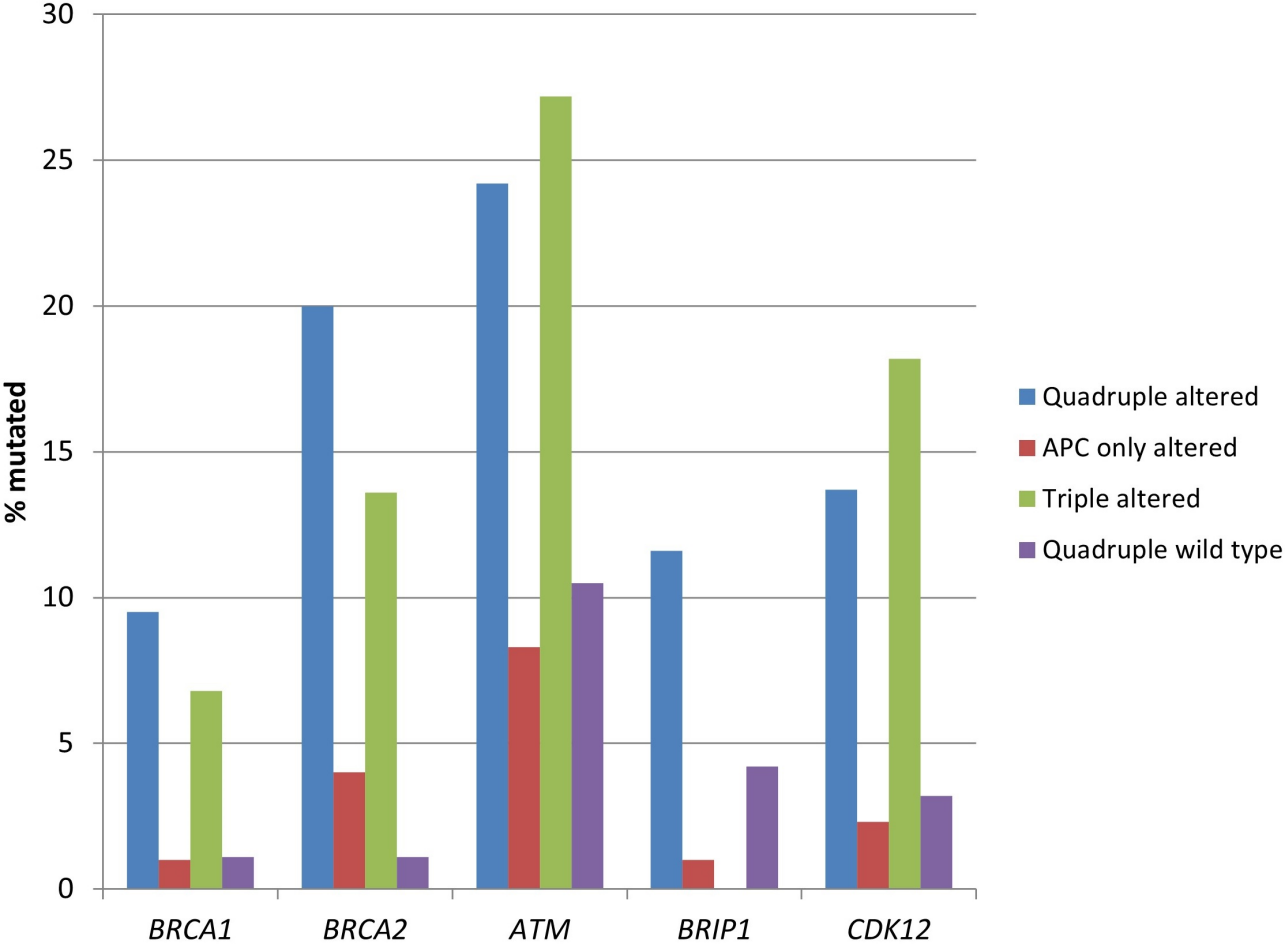
**Prevalence of mutations in genes encoding for proteins of the DNA damage response pathway in colorectal cancers with or without APC and other WNT/β-catenin/APC pathway alterations**. Data are from TCGA (the Cancer Genome Atlas). APC: adenomatous polyposis coli

DNA damage response genes, including *BRCA1*, *BRCA2*, *ATM*, and *CDK12* were more frequently mutated in the two groups with *RNF43*, *CTNNB1*, or *TCF7L2* alterations, both with (quadruple altered group) and without (triple altered group) *APC* alterations compared with the two groups without *RNF43*, *CTNNB1*, or *TCF7L2* alterations (Table 3 and Figure 5). *ATM* mutations had the highest prevalence of mutations in the two *RNF43*, *CTNNB1*, or *TCF7L2* altered groups with about one-fourth of the cases being mutated (Figure 5).

**Figure 6:**
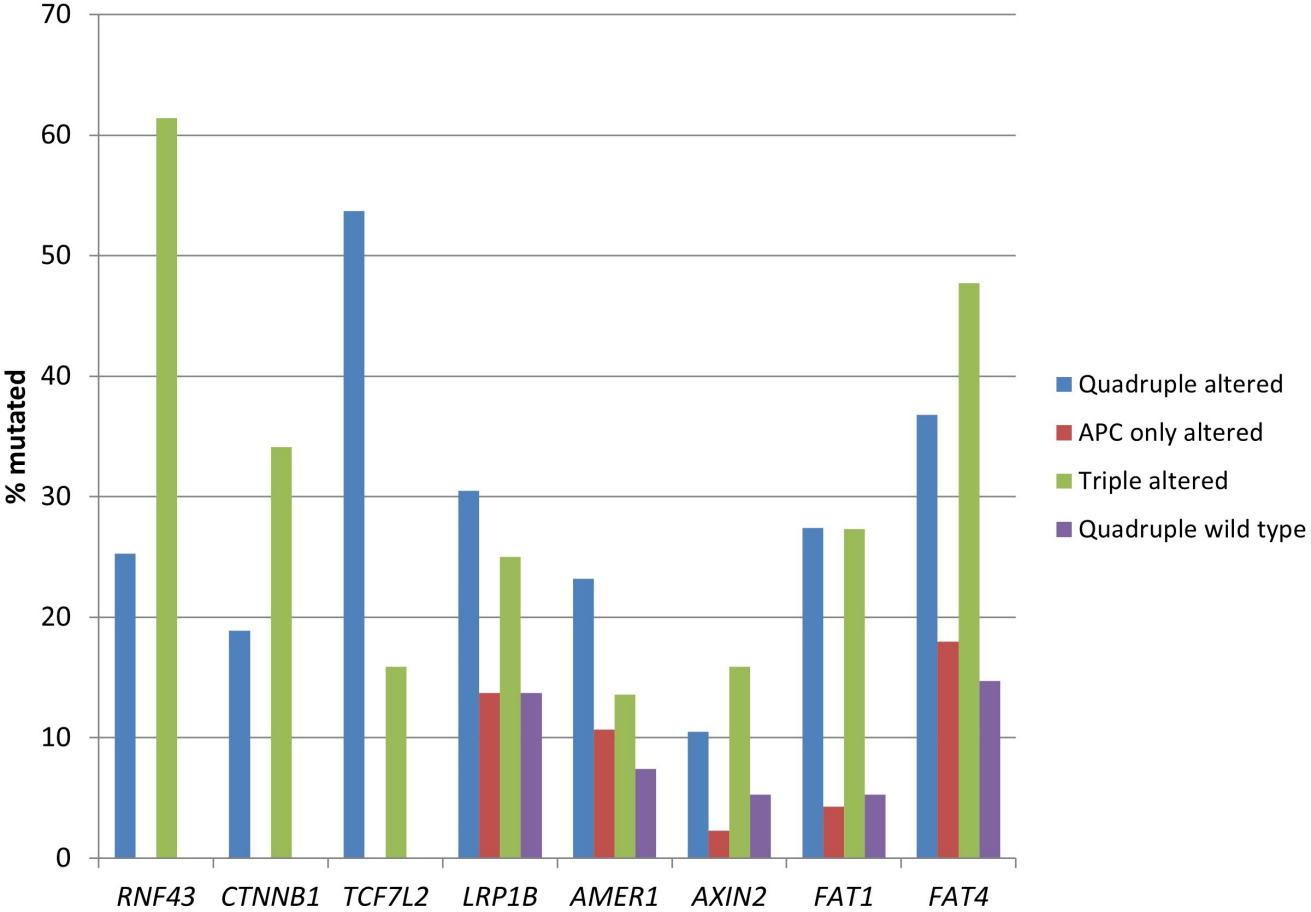
**Prevalence of mutations in genes encoding for other proteins of the WNT/β-catenin/APC pathway, besides *APC*, *RNF43*, *CTNNB1*, and *TCF7L2*, in colorectal cancers with or without APC and other WNT/β-catenin/APC pathway alterations.** Data are from TCGA (the Cancer Genome Atlas). APC: adenomatous polyposis coli

In addition to *RNF43*, *CTNNB1*, and *TCF7L2* alterations, the quadruple altered and triple altered groups displayed more frequent mutations in other components of the WNT/β-catenin/APC pathway, such as *LRP1B*, *AXIN2*, and the atypical cadherins *FAT1* and *FAT4* (Table 3 and Figure 6). *LRP1B* mutations were present in one-fourth of the cases, while *FAT1* and *FAT4* had an even higher prevalence of mutations in these groups.

### Cell lines comparison

Similar to colorectal cancer patient samples, *APC* mutations were the most common genomic alterations of the WNT/β-catenin pathway in the colorectal cancer cell line cohort of the CCLE collection consisting of 84 colorectal cancer cell lines. The three other genes of the pathway that are frequently mutated in colorectal cancer samples, *RNF43*, *CTNNB1*, and *TCF7L2*, were also frequently mutated in colorectal cancer cell lines, although less frequently than *APC*. Among *APC* mutated cell lines, 11 cell lines possessed pathogenic mutations in both *APC* and one or more of the three other genes of the pathway (the quadruple mutant group, Table 4). The cell lines with pathogenic *APC* mutations in the absence of mutations in *RNF43*, *CTNNB1*, or *TCF7L2* formed the *APC* only mutated group consisting of 13 cell lines. Nine colorectal cancer cell lines had mutations in one or more of the three other genes, in the absence of *APC* mutations (triple mutated cell lines, Table 4). A fourth group consisting of four cell lines were wild type for all four frequently mutated WNT/β-catenin pathway genes (*APC*, *RNF43*, *CTNNB1*, and *TCF7L2*: quadruple wild type cell lines) (Table 4).

**Table 4:** Colorectal cancer cell lines with or without APC and other WNT/β-catenin pathway mutations

**Cell line**	**DepMap ID**	**TMB**	**FGA**	**Ploidy**	**MSI status**
Quadruple mutated					
CL-34	ACH-000895	42.73333	0.149	1.98	NA
CW-2	ACH-000998	274.9667	0.0293	2	MSI
GP2d	ACH-000982	169.2	0.0418	2	NA
HCT-15	ACH-000997	259.6667	0.0269	2	MSI
KM12	ACH-000969	110.7	0.1304	2	MSI
LoVo	ACH-000950	77.5	0.2092	2.2	MSI
LS411N	ACH-000985	182.0667	0.2807	3.41	MSI
NCI-H630	ACH-002287	75.4	NA	NA	NA
SNU-C2A	ACH-000967	104.1667	0.1034	1.98	NA
SNU-C4	ACH-000959	73.46667	0.0337	1.99	NA
SNU-C5	ACH-000970	100.0333	0.0966	2.05	MSI
APC only mutated					
C75	ACH-001458	8.333333	NA	NA	NA
C80	ACH-001459	7.133333	NA	NA	NA
C84	ACH-001460	7.566667	NA	NA	NA
CACO2	ACH-000003	5.6	NA	NA	NA
COLO 201	ACH-000253	8.866667	0.38	2.96	MSS
DiFi	ACH-002233	7.2	NA	NA	MSS
GEO	ACH-002394	11.13333	NA	NA	MSS
HCC2998	ACH-001081	233.4333	NA	NA	MSS
MDST8	ACH-000935	25.86667	0.5583	2.12	MSS
SK-CO-1	ACH-000400	9.9	0.552	3.68	MSS
SNU-175	ACH-000989	183.8667	0.0927	2.05	MSI
SW1116	ACH-000489	12.03333	0.6339	2.76	MSS
SW1417	ACH-000236	8.5	0.5683	3.12	MSS
Triple mutated					
HCC-56	ACH-000467	12.96667	0.5288	2.94	NA
HCT 116	ACH-000971	106.5	0.082	2.06	MSI
LIM1215	ACH-001546	30.36667	NA	1.97	MSI
LS1034	ACH-000252	12.4	0.3562	3.11	MSS
NCI-H508	ACH-000360	10.73333	0.4813	2.31	MSS
NCI-H716	ACH-000491	14.1	0.6137	2.83	MSS
RKO	ACH-000943	114.6667	0.1421	2.15	MSI
SNU-407	ACH-000955	88.33333	0.0895	2.08	MSI
SNU-C2B	ACH-001199	86.2	NA	2.02	MSI
Quadruple wild type					
C10	ACH-001454	7.233333	NA	NA	NA
C99	ACH-001461	6.9	NA	NA	NA
CAR1	ACH-002345	7.833333	NA	2.77	MSS
LS513	ACH-000007	13.9	0.2636	2.28	MSS

TMB: tumor mutation burden; FGA: fraction genome altered; MSI: microsatellite instability; MSS: microsatellite stability; NA: not available; APC: adenomatous polyposis coli. Data are from the Cancer Cell Line Encyclopedia (CCLE)

Although data from some cell lines in the quadruple mutated group were not available, all cell lines of the group with data available were MSI high and had a high TMB (Table 4). All but one cell line was diploid and most had a low CIN as measured by the FGA. In contrast, and consistent with patient samples, all but one cell line in the group with *APC* only mutations were microsatellite stability (MSS), and had low TMB and high CIN. The group of cell lines with non-APC WNT/β-catenin pathway gene mutations (triple mutated group) was comprised of both MSS and MSI cell lines and a corresponding mixture of high and low TMB (Table 4). Lastly, the quadruple wild type group was comprised of cell lines with low TMB and MSS status. Pathogenic *TP53* mutations were present in 8 of the 11 (82.7%) cell lines of the quadruple mutated group, in 8 of the 13 (61.5%) cell lines of the APC only mutated group, in 6 of the 9 (66.7%) cell lines of the triple mutated group and in 1 of the 4 (25%) cell lines of the quadruple wild type group (Table 5). Classic codon 12 and 13 pathogenic *KRAS* mutations were present in 4 of the 11 (36.4%) quadruple mutated cell lines (one other cell line of the group had a mutation of unknown significance at codon 140). Pathogenic *KRAS* mutations were present in 7 of 13 (53.8%) cell lines of the APC only mutated group, in 5 of 9 (55.6%) cell lines of the triple mutated group, and in 1 of the 4 (25%) cell lines of the quadruple wild type group (Table 5). No cell lines in the four groups possessed mutations in the homologous *NRAS* gene, but 2 cell lines from the quadruple mutated group had homo-deletions of the *NRAS* locus at chromosome 1p13.2, an alteration that is rarely observed in colorectal cancer patient samples, with a prevalence of 0.7% in the colorectal cohort from TCGA.

**Table 5:** Alterations of frequently mutated genes of colorectal cancer in colorectal cancer cell lines with or without APC and other WNT/β-catenin pathway mutations

**Cell line**	***TP53***	***KRAS***	***NRAS***	***BRAF***	***PIK3CA***	***APC***	***RNF43***	***CTNNB1***	***TCF7L2***
Quadruple mutated									
CL-34	*S127P, *K382Nfs*40	WT	WT	*V600E	WT	*T1556Nfs*3, *E418*, R856C	WT	WT	*K468Sfs*23, L200I
CW-2	WT	P140H	WT	WT	P283S	*S1465Wfs*3, *R302*, G470R, A528V, E2737G, I2756V	WT	R582Q	*C469Vfs*8
GP2d	WT	*G12D	WT	T529A	*H1047L	*T1445Lfs*27, L2384I, S2562G, HD	S771T, D628G	D755V	*W156*
HCT-15	*S241F, *X367_splice	*G13D	WT	WT	*E545K, *D549N	*I1417Lfs*2, *R2166*, R727M, K993N, K1561N, E2550Q, I1779M	*G659Vfs*41, L214M	WT	WT
KM12	*H179R, *V73Wfs*50	WT	WT	A712T, A404Cfs*9	WT	*N1818Kfs*2, G471E	*G659Vfs*41	SETD5 fusion	WT
LoVo	WT	*G13D	WT	WT	WT	*R1114*, *M1431Cfs*42, R2816Q	WT	*R535Q	*K468Sfs*23
LS411N	*Y126*	WT	WT	*V600E	WT	*T1556Nfs*3, *Q789*	*G659Vfs*41	WT	WT
NCI-H630	*R342*	WT	WT	WT	WT	*Q1367*, V1173M	WT	WT	*K468Sfs*23
SNU-C2A	*R273C, *R273H, *R273Y	*G12D	HD	WT	D725G	*K2051Efs*9	*R337*, C275Wfs*143, R389C	HD	*K468Sfs*23
SNU-C4	*G245S	WT	HD	D22N	*E545G, V71I	*F801Lfs*19, *T1556Lfs*9, H325R	*R225Afs*194	WT	*K468Sfs*23
SNU-C5	*R248W, *V218L	WT	WT	*V600E	*H1047R	*N1792Kfs*7	*G659Vfs*41	WT	WT
*APC* only mutated									
C75	*R249S	WT	WT	WT	WT	*Q1204*, *S943Qfs*12, *L1488Ffs*23	WT	WT	WT
C80	*Q52*	*A146V	WT	WT	WT	*L629*	WT	WT	WT
C84	*R342*	*G12A	WT	WT	WT	*R1450*, *R283*, *R640W	WT	WT	WT
CACO2	*C135F, *E204*	WT	WT	WT	WT	*Q1367*	WT	G245A	WT
COLO 201	WT	WT	WT	*V600E	WT	*T1556Nfs*3	WT	WT	WT
DiFi	*K132R	WT	WT	WT	WT	*E1151*, *E443Afs*16	WT	WT	WT
GEO	WT	*G12A	WT	WT	WT	*E1536*, *C344Vfs*110	WT	WT	WT
HCC2998	*R213*	*A146T	WT	WT	WT	*R1450*, *L665*, I2167S, S1864Y, N2720K, R168I	WT	WT	WT
MDST8	WT	WT	WT	*V600E, *V600K, *V600M	WT	*T1556Nfs*3	WT	WT	WT
SK-CO-1	WT	*G12V, AMPL	WT	AMPL	WT	*F1089fs*37	WT	WT	WT
SNU-175	WT	*A59T	WT	NA	NA	*R232*, *N1455fs*18, *G1499*, A199V	WT	WT	WT
SW1116	*A159D	*G12A	WT	NA	AMPL	*Q264*, *Q1429Hfs*41	WT	WT	WT
SW1417	*C238Hfs*21	WT	WT	*V600E, AMPL	NA	*R1450*	WT	WT	WT
Triple mutated									
HCC-56	*R196P	*G12V	WT	WT	WT	WT	WT	WT	*X387_splice
HCT 116	WT	*G13D	WT	WT	*H1047R	WT	*R117Afs*41	*S45del	Fusion
LIM1215	WT	WT	WT	WT	WT	WT	WT	*T41A, Q177P	NA
LS1034	*G245S, HD	*A146T, AMPL	WT	WT	WT	WT	WT	WT	*R39Gfs*4
NCI-H508	*R273H	WT	WT	*G596R	*E545K, AMPL	WT	WT	WT	Fusion, HD
NCI-H716	*E224D	R97I	WT	WT	WT	WT	*H472Qfs*30	WT	WT
RKO	WT	WT	WT	*V600E	*H1047R	WT	*G659Vfs*41, H549N	WT	WT
SNU-407	*S90Pfs*33	*G12D	WT	R726C	*H1047R	WT	WT	*T41A	*K468Sfs*23, A549T, A419V
SNU-C2B	*R273Y	*G12D	WT	WT	D725G	WT	*C275Wfs*143	WT	*K468Sfs*23
Quadruple wild type									
C10	WT	WT	WT	WT	WT	WT	WT	WT	WT
C99	WT	WT	WT	WT	WT	WT	WT	WT	WT
CAR1	*V272M	WT	WT	WT	WT	WT	WT	WT	WT
LS513	WT	*G12D	WT	E204L, E204V, E204*	WT	WT	WT	WT	WT

AMPL: amplified; HD: Homodeleted; WT: wild type; NA: not available; APC: adenomatous polyposis coli. * before a mutation denotes oncogenic. Data are from the Cancer Cell Line Encyclopedia (CCLE)

**Figure 7:**
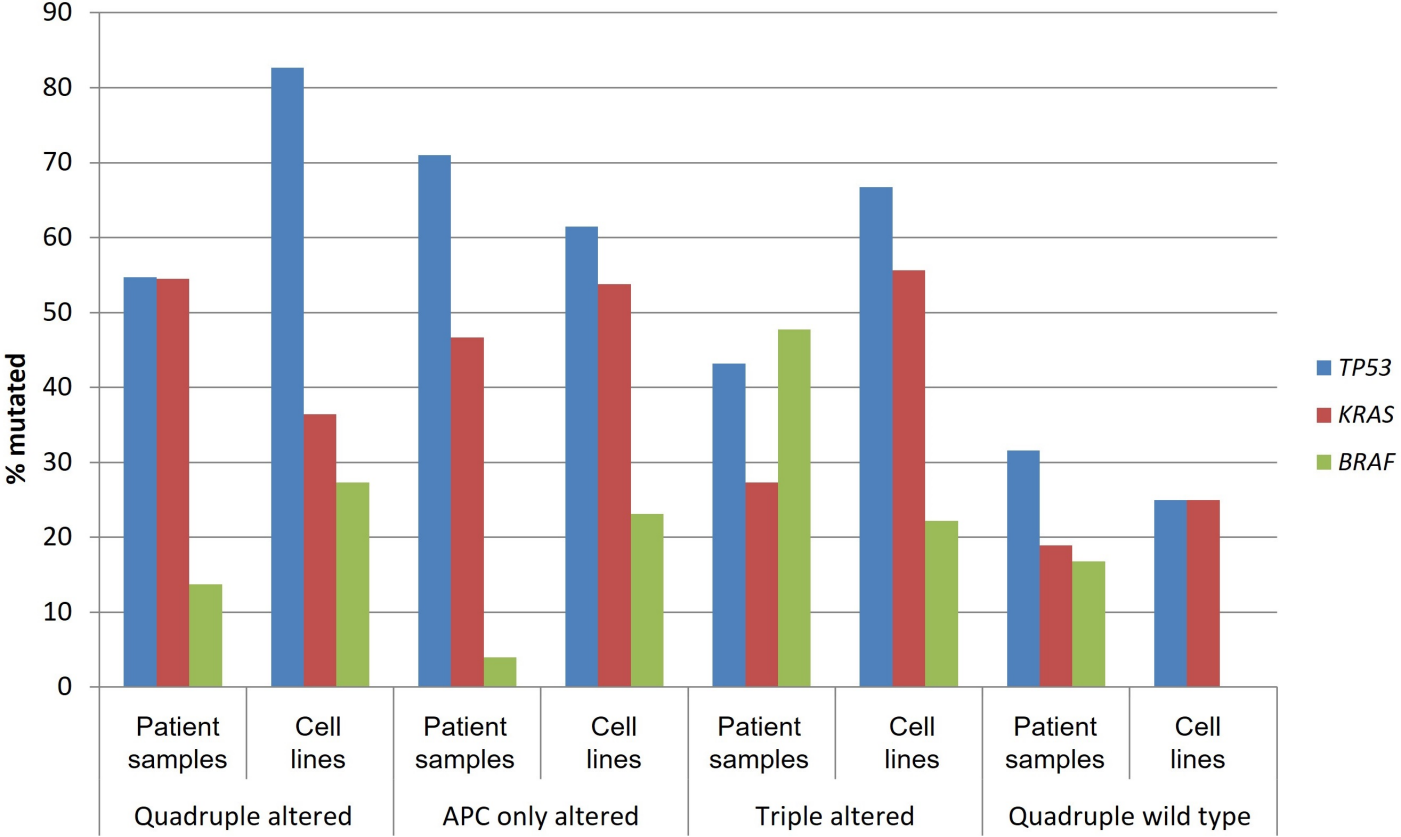
**Prevalence of mutations in *TP53*, *KRAS*, and *BRAF* in patient samples and corresponding cell lines from colorectal cancers with or without APC and other WNT/β-catenin/APC pathway alterations**. Data for patient samples are from TCGA (the Cancer Genome Atlas) and for cell lines from the Cancer Cell Line Encyclopedia. APC: adenomatous polyposis coli

V600E or other pathogenic *BRAF* mutations were observed in 3 of 11 (27.3%) quadruple mutated cell lines, in 3 of 13 (23.1%) cell lines of the APC only mutated group, in 2 of 9 (22.2%) cell lines of the triple mutated group, and no cell lines of the quadruple wild type group (Table 5). Overall, the colorectal cancer cell lines in the four groups capture to a significant degree, albeit with some variability, the corresponding prevalence of the pathogenic mutations in *TP53*, *KRAS*, and *BRAF* in patient samples (Figure 7). For example, pathogenic *BRAF* mutations were more frequent in cell lines of the quadruple altered and APC only altered groups than the corresponding patient samples, while in the triple altered group, where pathogenic *BRAF* mutations were most prevalent in patient samples, corresponding cell lines had a lower frequency of *BRAF* mutations (Figure 7).

### Drug sensitivities in vitro

Comparison of the sensitivities of the four groups of colorectal cancer cell lines to representative porcupine inhibitors, tankyrase inhibitors, and EGFR inhibitors was performed using data from the GDSC project. Although the small number of in vitro assayed cell lines prevented a formal statistical comparison from reaching significance, the two quadruple cell lines with data available seemed to be more sensitive to two of the three porcupine inhibitors tested, all three tankyrase inhibitors with data and to be more sensitive to the EGFR inhibitor erlotinib than cell lines from the other groups (Table 6).

**Table 6:** **Sensitivities (mean IC_50_**, μ**M) of colorectal cancer cell lines with or without APC and other WNT/β-catenin pathway mutations to porcupine inhibitors, tankyrase inhibitors, and EGFR inhibitors**

**Groups**	**Porcupine inhibitors**			**TNKRS inhibitors**				**EGFR inhibitors**	
	**LGK974**	**IWP-2**	**Wnt-C59**	**AZ6102**	**MN-64**	**WIKI4**	**XAV939**	**Cetuximab**	**Erlotinib**
Quadruple mutated (*n* = 6)	138.5 (77.9)	33.31 (16.56)	150.8 (136.6)	25.39 (38.13)	211.7 (84.2)	79.01 (64.09)	156.1 (76.8)	543.5 (367.2)	21.01 (33.74)
*APC* only mutated (*n* = 7)	130.04 (91.3)	38.72 (28.7)	168.02 (164.6)	29.79 (48.04)	218.3 (189.2)	45.13 (22.23)	175.2 (113.5)	418.6 (494.4)	30.67 (57.39)
Triple mutated (*n* = 7)	134.57 (94.1)	37.01 (32.9)	154.5 (122.2)	15.31 (10.66)	168.1 (135.8)	66.79 (52.63)	110.5 (40.7)	657.1 (349.4)	22.75 (16.56)
Quadruple wild type (*n* = 2)	64.05 (30.05)	31.6 (0.91)	135.5 (127.7)	4.2 (2.82)	110.5 (68.3)	NA	51.61 (62.8)	838.9 (322.4)	5.51 (4.32)
ANOVA	*F* = 0.4, *P* = 0.74	*F* = 0.06, *P* = 0.97	*F* = 0.03, *P* = 0.99	*F* = 0.38, *P* = 0.76	*F* = 0.4, *P* = 0.75	*F* = 0.82, *P* = 0.45	*F* = 1.5, *P* = 0.22	*F* = 0.69, *P* = 0.56	*F* = 0.23, *P* = 0.87

Standard deviations (SD) are shown in parentheses. Data are from the Genomics of Drug Sensitivity in Cancer (GDSC) project. NA: not available; APC: adenomatous polyposis coli

RNAi and CRISPR arrays showed that knock out or knock down of at least one of the four WNT/β-catenin pathway genes of interest (*APC*, *RNF43*, *CTNNB1*, and *TCF7L2*) in all but one of the cell lines of the quadruple mutated group produced significant growth delay gene effect (Z-score < –2.5). Most cell lines of the group were sensitive to the knock out or knock down of *CTNNB1* (Tables 7 and 8). In the group of *APC* only altered colorectal cancer cell lines, knock out or knock down of *APC* had in general no significant effect. In contrast, growth delay was observed with RNAi knock down and even more with CRISPR knock out of *CTNNB1* and *TCF7L2*, which are downstream of the pathway defect in these cells (Tables 7 and 8). Some cell lines of the triple altered group were also sensitive to knock out or knock down of *CTNNB1* and *TCF7L2*, but others were resistant to all four genes knock out or knock down. Data for quadruple wild type cell lines were limited and were available for one cell line for RNAi and three cell lines for CRISPR (Tables 7 and 8). Cell line LS513 displayed sensitivity to knock out and knock down of *TCF7L2* (Z-score < –2.5), while cell lines C10 and C99 appeared to be resistant to knock down of all four genes.

**Table 7:** RNA interference (RNAi) in colorectal cancer cell lines with or without APC and other WNT/β-catenin pathway mutations

**Cell line**	***APC***			***RNF43***			***CTNNB1***			***TCF7L2***		
	**Gene effect**	**Z-score**	**Rank**	**Gene effect**	**Z-score**	**Rank**	**Gene effect**	**Z-score**	**Rank**	**Gene effect**	**Z-score**	**Rank**
Quadruple mutated												
CL-34	–0.58	–2.67	8	–0.18	–0.57	1,040	–1.87	–3.01	4	–0.3	–1.12	275
CW-2	–0.18	–0.89	2,285	0.3	2.7	7,993	–4.71	–8.3	3	–0.61	–2.66	354
GP2d	–0.82	–3.78	4	–0.15	–0.4	3,379	–2.06	–3.36	7	–0.16	–0.41	3,310
HCT-15	0.05	0.23	5,480	–0.01	0.55	6,368	–1.94	–3.14	97	0.06	0.71	6,728
KM12	–0.28	–1.3	790	–0.2	–0.67	2,741	–1.8	–2.89	28	–0.11	–0.17	5,904
LoVo	–0.42	–1.93	254	–0.19	–0.61	3,380	–1.66	–2.63	45	–0.14	–0.3	5,135
LS411N	–0.51	–2.35	159	–0.02	–0.52	8,767	–2.18	–3.6	21	0.02	0.51	8,707
SNU-C2A	0.54	2.46	12,975	–0.06	0.24	8,690	–0.17	0.15	8,195	–0.28	–1	2,370
SNU-C4	–0.38	–1.79	101	–0.16	–0.46	1,606	–2.04	–3.34	16	–0.27	–0.94	550
APC only mutated												
CACO2	0.08	0.34	10,937	–0.16	–0.41	6,307	–0.91	–1.23	2,220	0.11	0.96	13,729
COLO 201	–0.18	–0.85	2,324	0.009	0.73	5,956	–2	–3.2	107	–1.35	–6.34	5
MDST8	0.08	0.36	4,453	0.07	1.17	6,420	–0.64	–0.73	1,816	–0.16	–0.43	2,428
SK-CO-1	–0.72	–3.31	28	0.04	0.99	11,450	–1.65	–2.6	64	–0.47	–1.97	177
SW1417	–0.18	–0.86	1,711	–0.19	–0.66	2,323	0.09	0.65	9,553	–0.3	–1.12	1,075
Triple mutated												
HCT 116	–0.05	–0.27	4,669	–0.2	–0.69	2,279	–0.56	–0.58	2,766	–0.28	–0.99	1,297
NCI-H508	–0.11	–0.53	3,510	–0.26	–1.08	1,563	–1.13	–1.64	509	–0.72	–3.23	10
NCI-H716	0.05	0.23	7,744	0.04	1	11,047	–0.12	0.23	7,758	0.1	0.92	10,816
RKO	0.07	0.29	8,335	–0.15	–0.36	3,192	–0.05	0.36	8,962	0.03	0.56	10,424
SNU-407	0.2	0.89	6,985	–0.17	–0.48	3,041	–2.84	–4.83	7	–0.68	–3.03	68
Quadruple wild type												
LS513	–0.28	–1.32	1,188	–0.02	0.47	8,506	–1.63	–2.57	107	–0.63	–2.75	66

Data were from project Achilles, Drive, Marcotte DEMETER2. The Z-score was computed as the gene effect minus the mean across cell line models divided by the standard deviation (SD). Not all cell lines of the original cohorts had data available in the RNAi arrays. APC: adenomatous polyposis coli

**Table 8:** Gene knocks out with CRISPR in colorectal cancer cell lines with or without APC and other WNT/β-catenin pathway mutations

**Cell line**	***APC***			***RNF43***			***CTNNB1***			***TCF7L2***		
	**Gene effect**	**Z-score**	**Rank**	**Gene effect**	**Z-score**	**Rank**	**Gene effect**	**Z-score**	**Rank**	**Gene effect**	**Z-score**	**Rank**
Quadruple mutated												
HCT-15	–0.27	0.23	10,419	–0.07	–0.7	3,831	–1.36	–3.08	122	0.09	0.75	13,906
KM12	–0.21	0.45	12,777	0.19	1.67	17,604	–1.42	–3.22	15	–0.25	–0.65	3,705
LoVo	–0.44	–0.42	5,884	–0.12	–1.11	2,469	–1.16	–2.54	189	–0.47	–1.55	1,284
LS411N	–0.13	0.76	12,095	0.03	0.25	9,534	–2.51	–6.15	19	–0.6	–2.09	1,222
SNU-C4	–0.61	–1.06	2,218	0.13	1.13	15,995	–1.72	–4.04	11	–1	–3.69	21
SNU-C5	–0.35	–0.08	7,663	–0.04	–0.42	5,354	–0.32	–0.27	6,313	–0.22	–0.55	4,629
APC only mutated												
C75	–0.13	0.75	12,681	0.21	1.9	16,539	–1.2	–2.64	477	–0.92	–3.37	155
C80	0.13	1.82	16,873	–0.006	–0.09	8,039	–1.12	–2.42	456	–0.71	–2.5	401
C84	–0.76	–1.66	1,204	0.06	0.53	11,781	–2.11	–5.09	4	–0.79	–2.84	174
COLO 201	–0.28	0.18	10,692	0.06	0.56	14,017	–1.9	–4.52	14	–0.91	–3.32	37
DiFi	–0.05	1.06	13,404	0.1	0.9	12,728	–1.08	–2.31	924	–0.6	–2.09	1,219
HCC2998	–0.35	–0.07	7,755	–0.02	–0.26	6,743	–1.58	–3.65	195	–0.69	–2.44	746
MDST8	–0.39	–0.24	6,913	0.06	0.51	12,398	–0.89	–1.82	777	–0.05	0.13	9,717
SW1116	–0.43	–0.37	4,910	0.03	0.32	12,360	–0.35	–0.36	4,978	–0.74	–2.62	73
Triple mutated												
HCC-56	0.05	1.51	16,795	0.1	0.88	14,525	–0.53	–0.84	3,428	–0.71	–2.52	182
HCT 116	–0.12	0.81	13,695	0.05	0.41	11,203	–0.61	–1.07	2,413	–0.52	–1.75	953
LS1034	0.28	2.38	17,489	0.23	2.03	17,082	–1.71	–4.01	43	–1.03	–3.78	63
NCI-H716	–0.34	–0.05	7,958	0.04	0.41	10,878	0.01	0.61	12,069	0.09	0.72	12,712
RKO	0.01	1.36	17,773	0.07	0.66	15,835	–0.06	0.4	13,860	–0.02	0.23	12,188
Quadruple wild type												
C10	–0.72	–1.51	1,919	–0.01	–0.14	7,219	–0.05	0.44	10,809	–0.08	0.01	8,181
C99	–0.25	0.3	10,691	0.02	0.15	9,788	–1.09	–2.35	563	–0.52	–1.75	1,355
LS513	–0.18	0.57	12,725	–0.09	–0.85	3,479	–1	–2.11	496	–0.77	–2.75	176

Data are from the DepMap, Public 24Q2+Score, and Chronos projects. The Z-score was computed as the gene effect minus the mean across cell line models divided by the standard deviation (SD). Not all cell lines of the original cohorts had data available in the CRISPR arrays. APC: adenomatous polyposis coli

## Discussion

The WNT/β-catenin/APC pathway provides physiologic signals for the maintenance of the stem cells in the intestinal crypts [31]. Paneth cells in the crypts as well as mesenchymal cells surrounding the crypt epithelium secrete ligands of the pathway and lead to the physiologic activation of cell proliferation of the stem cells through cyclin D induction. Activation signals and the activity status of the WNT/β-catenin/APC pathway decrease with increasing distance from the bottom of the crypt and along the intestinal villi. In contrast, the gradient of activity of the bone morphogenic protein (BMP) signaling pathway, which counteracts WNT/β-catenin/APC pathway activity and promotes differentiation, increases from the bottom of the crypt and towards the tip of villi [31]. In addition to WNT/β-catenin activity, the crypt micro-environment displays increased activity of the EGFR pathway. EGFR signals are critical for the proliferation of intestinal stem cells, as well as for orchestrating metabolic processes [32, 33]. In colorectal cancer, the WNT/β-catenin/APC pathway is aberrantly activated through mutations in *APC* and less frequently mutations in other pathway genes, which result in autonomous signaling without external signals from the micro-environment through receptor ligation [34]. The deregulated activity of the pathway endows cancer cells with properties of stem cells such as increased self-renewal, increased proliferation potential, and drug resistance [35]. Therefore, inhibition of the aberrantly activated pathway could be a rational target for colorectal cancer therapies.

In the current investigation, the genomic landscape of sub-sets of colorectal cancers based on the presence of alterations in four key WNT/β-catenin/APC pathway genes has been established using data from the colorectal cancer cohort of TCGA. Therapeutic sensitivities to drugs inhibiting the pathway were explored in cell lines, according to the presence of WNT/β-catenin/APC pathway alterations. It was discovered that colorectal cancers possessed significant differences in their genomic profile dependent on whether the WNT/β-catenin/APC pathway was activated through alterations in *APC* or through alterations in three alternative genes of the pathway, *RNF43*, *CTNNB1*, and *TCF7L2*. Colorectal cancers with alterations in *RNF43*, *CTNNB1*, and *TCF7L2* without *APC* alterations presented more commonly MSI and a high TMB. In addition, cancers with alterations in *RNF43*, *CTNNB1*, and *TCF7L2* displayed high rates of mutations in receptor tyrosine kinases, MMR-associated genes and DDR-associated genes, independently of the presence or absence of concomitant *APC* alterations. Cell lines of colorectal cancer origin partially recapitulated the landscape of WNT/β-catenin/APC pathway alterations, being more frequently mutated for *APC* than the other genes of the pathway, but some features of the patient sample groups were not well represented. For example, cell lines of the quadruple altered group were mostly MSI high, while MSI high cases comprised 22.1% of the patient samples in the quadruple altered group. In addition, the prevalence of *BRAF* mutations in triple altered cell lines was not higher than the prevalence in quadruple altered and *APC* only altered cell lines, in contrast to patient samples where the prevalence of *BRAF* mutations in triple altered cases were significantly higher than in the other groups. This divergence of the molecular landscape between patient samples and cell lines needs to be considered when evaluating studies based on cell lines. Studies of in vitro sensitivity to inhibitors of the WNT/β-catenin pathway and EGFR inhibitors were limited by the small number of cell lines, but a numerical greater sensitivity of wild type cell lines for the key genes of the WNT/β-catenin/APC pathway to several of these inhibitors was observed. RNAi and CRISPR arrays suggested the sensitivity of quadruple mutant cell lines to knock down or knock out of *APC* or other genes of the pathway, while *APC* only altered cell lines were mostly resistant to *APC* knock down or knock out but sensitive to knock down or knock out of other genes of the pathway, especially *CTNNB1*, encoding for β-catenin. These data confirm that the specific alterations in the WNT/β-catenin/APC pathway modulate sensitivities to additional interventions affecting pathway activity.

Drugs inhibiting two types of enzymes that function in the activation of the WNT/β-catenin pathway, porcupine and tankyrases 1 and 2 have been included in the panel of drugs tested in the GDSC [19]. Porcupine is a membrane-bound *o*-acyltransferase which is a crucial enzyme for the lipidation of ligands of the WNT pathway, a process that is a prerequisite for their secretion and subsequent receptor binding for signaling [36]. Inhibitors of the enzyme have progressed to early-phase clinical trials. The inhibitor WNT974 was studied in combination with encorafenib and cetuximab in a phase Ib/II trial of patients with *BRAF* V600E mutated, *KRAS* wild type metastatic colorectal cancer [37]. Selection for alterations in the WNT/β-catenin pathway was not performed in this trial. The triple combination showed a low overall response rate of 10%. In addition, serious adverse events including several patients with bone fractures, as well as hypercalcemia and pleural effusions were observed. Fractures are an on-target adverse effect, as WNT signaling is involved in trabecular bone formation and prevention of resorption [38]. Therefore, more advanced trial development of the drug was interrupted.

Tankyrases 1 and 2, also called PARP5A and PARP5B, are enzymes of the PARP family that perform the enzymatic PARylation promoting subsequent axin ubiquitination and degradation, and, as a result, β-catenin stabilization [39]. Phosphorylation of axin by Casein kinase 1 is a negative regulator of the WNT/β-catenin signaling. This phosphorylation of axin involves the same site as PARylation and prevents PARylation by tankyrases [40]. Moreover, tankyrases have additional enzymatic targets, including *PTEN*, angiomotins, and DNA-associated protein kinase (DNA-PK), through which they promote the activity of PI3K/AKT pathway, the YAP pathway and DNA repair by the error-prone non-homologous end joining (NHEJ) mechanism. Therefore, discovery of clinical-grade tankyrase inhibitors has been prioritized as a potential anti-cancer therapeutic strategy with several compounds examined in pre-clinical studies [39, 41]. For example, the inhibitors XAV939 and IWR-1 have been effective in inhibiting β-catenin transcription activity by promoting its degradation in colorectal cancer cells [42].

Blocking the WNT/β-catenin pathway at the level of ligand secretion or enhancing β-catenin destruction in colorectal cancers with *APC* mutations may not be the most effective strategies, as deregulated signaling is independent of pathway receptor engagement with ligands and the destruction complex assembly is already defective. Approaches to block the WNT/β-catenin pathway in cancers with *APC* mutations, such as the great majority of colorectal cancers, would require inhibitors of processes downstream of the defective β-catenin destructive complex. In this vein, inhibitors of the TRAF2 and NCK interacting protein kinase (TNIK), which regulates the TCF7L2/β-catenin transcription complex, and inhibitors of the interaction of β-catenin with acetyltransferase CBP have been discovered [43]. *TCF7L2* is a target for phosphorylation by kinase TNIK and this phosphorylation is crucial for transcription of target genes of the TCF7L2/β-catenin complex [44]. TNIK is also involved in other cancer-associated processes through alternative targets including AKT signaling, autophagy and the epithelial to mesenchymal transition, and modulation of these processes would also have to be considered when targeting the kinase. Moreover, it would be of interest to investigate how pathogenic or non-pathogenic *TCF7L2* mutations affect the sensitivity of cancer cells to TNIK inhibitors. In contrast to cancers with *APC* or other defects in the β-catenin destruction complex, colorectal cancers with *RNF43* mutations may retain WNT pathway ligands dependence and may as a result be better targets for porcupine and tankyrase inhibitors [45].

In conclusion, therapeutic inhibition of the WNT/β-catenin pathway in colorectal cancers, which possess in their majority *APC* mutations but also present, in a significant minority of cases, mutations in other key proteins of the pathway, needs to be developed with these specific alterations taken into consideration. The specific alteration of the WNT/β-catenin pathway present in a given colorectal cancer is of significant importance given that different proteins of the pathway play additional roles in cancer-associated cellular functions independent of WNT signaling. For example, *APC* has function in mitotic spindle integrity and cell cycle control [46]. In another example, β-catenin has roles, beyond the WNT pathway, in cell adhesion and transcription cooperation with other pathways [47]. Data presented here may suggest that inhibitors that act at or upstream of the β-catenin destruction complex are more effective for the minority of colorectal cancers with no alterations in the main proteins of the WNT/β-catenin pathway. A rational development strategy for these inhibitors selecting patients according to the presence or absence of target pathway alterations may increase the chances of therapeutic success.

## Abbreviations

APC: adenomatous polyposis coli
AS: aneuploidy score
CCLE: Cancer Cell Line Encyclopedia
CIN: chromosomal instability
CRISPR: Clustered Regularly Interspaced Short Palindromic Repeats
GDSC: Genomics of Drug Sensitivity in Cancer
GISTIC: Genomic Identification of Significant Targets in Cancer
MMR: mismatch repair
MSI: microsatellite instability
RNAi: RNA interference
TCGA: the Cancer Genome Atlas
TMB: tumor mutation burden
TNIK: TRAF2 and NCK interacting protein kinase

## Declarations

### Availability of data and materials

The datasets analyzed for this study can be found in the Cancer Genome Atlas [http://www.cancer.gov/ccg/]; Cancer Cell Line Encyclopedia [http://www.sites.broadintitute.org/ccle/]; Genomics of Drug Sensitivity in Cancer (GDSC) project [http://www.cancerrxgene.org]; project Achilles, Drive, Marcotte DEMETER2 [http://www.depmap.org/portal/achilles]; DepMap, Public 24Q2+Score, and Chronos projects [http://www.depmap.org].

### Copyright

© The Author(s) 2025.
